# Supplementary material for: GiOPARK Project: The Genetic Study of Parkinson’s Disease in the Croatian Population
Source: Genes (Basel). 2024 Feb 19;15(2):255. doi: 10.3390/genes15020255 (PMC10888376; doi:10.3390/genes15020255)
Supplement: Supplementary file 1 [file genes-15-00255-s001.zip › genes-2872356-supplementary.pdf]

Table S1. Gene panels used in the study.

Parkinson's disease panel v1.68	ATP13A2, ATP1A3, C19orf12, CSF1R, DCTN1, DNAJC6, FBXO7, FTL, GBA, GCH1, GRN, LRRK2, LYST, MAPT, OPA3, PANK2, PARK7, PINK1, PLA2G6, PRKN, PRKRA, PTRHD1, RAB39B, SLC30A10, SLC39A14, SLC6A3, SNCA, SPG11, SPR, SYNJ1, TH, TUBB4A, VPS13A, VPS35, WDR45, CHCHD2, TAF1 and NR2A4.
Neurodegenerative disease panel v2.4	ABCD1, AFG3L2, ALS2, ANG, ANXA11, APP, ARSA, ATP13A2, ATP1A3, ATP7B, AUH, C19orf12, CACNA1G, CCNF, CHCHD10, CHCHD2, CHMP2B, CLCN2, CLN6, COASY, CP, CSF1R, CTSF, CYP27A1, CYP7B1, DARS2, DCTN1, DNAJC5, DNAJC6, DNMT1, EIF2B1, EIF2B2, EIF2B3, EIF2B4, EIF2B5, ELOVL4, EPM2A, FBXO7, FIG4, FTL, FUS, GCH1, GFAP, GRN, HEXA, HEXB, HNRNPA1, HTRA1, ITM2B, KCNC3, KCND3, MYORG, KIF5A, LRRK2, LYST, MAPT, NHLRC1, NOTCH3, NPC1, NPC2, OPTN, PANK2, PARK7, PDGFB, PDGFRB, PFN1, PINK1, PLA2G6, PRKN, PRNP, PSEN1, PSEN2, RNF216, SETX, SLC20A2, SNCA, SOD1, SPAST, SPG11, SQSTM1, SYNJ1, TARDBP, TBK1, TMEM240, TREM2, TTC19, TYROBP, UBQLN2, VAPB, VCP, VPS13A, VPS35, WDR45, XPR1, AP5Z1, ARHGEF28, ATP2B3, ATP6AP2, CCDC88C, CIZ1, COQ2, DAO, DNAJC13, EIF4G1, ERBB4, EWSR1, GBA, GCDH, GIGYF2, HNRNPA2B1, MARS2, MATR3, NEK1, NR4A2, PRKRA, PRPH, SIGMAR1, SLC30A10, SNCB, SS18L1, TAF1, TUBA4A, TUBB4A and VPS13C.

Table S2. List including variants of unknown significance, likely benign and benign variants.

Gene (transcript)	Variant	Class	Tot.	EO	FO	SO	Patient ID
GBA (NM_000157.4)	c.882T>G	VUS	1	0	0	1	PT074
ITM2B (NM_021999.5)	c.711T>G	VUS	1	0	0	1	PT068
LRRK2 (NM_198578.4)	c.3705G>T	VUS	1	1	0	0	PT022
	c.2576G>T	VUS	1	0	1	0	PT006
	c.356T>C	LB	1	1	0	0	PT107

	c.1918G>T	LB	1	1	0	0	PT049
ATP13A2 (NM_001141974.3)	c.58A>C (HET)	VUS	1	1	0	0	PT065
	c.3188C>A (HET)	VUS					
	c.3278T>C (HET)	VUS	1	0	1	0	PT010
	c.1744G>A (HET)	VUS					
	c.1634G>A (HET)	VUS	1	0	0	1	PT077
	C25G>T (HET)	VUS	1	0	1	0	PT035
	c.2836A>T (HET)	LB	2	0	0	2	PT031, PT081
	c.3361A>T (HET)	B	2	0	0	2	PT017, PT097
SNCA (NM_000345.3)	c.44T>C	VUS	1	0	1	0	PT038
VPS35 (NM_018206.6)	c.1327A>G	VUS	1	1	0	0	PT020
CHCHD2 (NM_001320327)	c.359A>G	VUS	1	0	1	0	PT055
ARSA (NM_000487.6)	c.542T>G	P (HET)	1	1	0	0	PT009
DNAJC13 (NM_001329126.2)	c.6148G>A	LB	1	0	1	0	PT035
	c.3569C>T	VUS	1	0	0	1	PT031
	c.3887A>G	B	2	0	0	2	PT052, PT125
	c.2627C>G	VUS	1	1	0	0	PT134
	c.424C>G	LB	1	0	0	1	PT022
EIF4G1 (NM_001194946.2)	c.4567T>G	VUS	1	1	0	0	PT142
	c.2651G>A	VUS	1	0	0	1	PT106
	c.1667C>T	VUS	1	0	0	1	PT076
	c.4085T>C	LB	1	0	0	1	PT047
GIGYF2 (NM_001103147.2)	c.281T>C	VUS	1	0	0	1	PT043
	c.1433A>C	B	2	0	0	2	PT100, PT116

	c.3575A>G	LB	1	1	0	0	PT007
	c.2447G>A	LB	1	0	0	1	PT137
CCDC88C (NM_001080414.4)	c.5231C>T	VUS	1	1	0	0	PT061
	c.5836C>T	LB					
	c1867G>T	VUS	1	0	0	1	PT119
DNMT1 (NM_001130823)	c.935A>G	VUS	1	0	0	1	PT110
	c.2728G>T	B	1	0	0	1	PT079
GRN (NM_002087.3)	c.229G>A	VUS	1	0	0	1	PT031
	c.970G>A	LB	1	0	0	1	PT111
KCNC3 (NM_004977.3)	c.1706C>G	VUS	1	0	0	1	PT056
	c.811C>T	VUS	1	0	0	1	PT003
	c.2197C>A	LB	1	1	0	0	PT134
ERBB4 (NM_005235.3)	c.794C>T	VUS	1	1	0	0	PT020
	c.847T>C	VUS	1	0	0	1	PT040
GCDH (NM_000159.3)	c.1262C>T (HET)	VUS	1	1	0	0	PT019
GCH1 (NM_000161.3)	c.206C>T	VUS	1	0	0	1	PT004
GFAP (NM_001363846)	c.85C>T	LB	1	0	1	0	PT050
HRPNPA1 (NM_031157.4)	c.692G>A	VUS	1	0	0	1	PT135
KIF5A (NM_004984.4)	c.2263G>A	VUS	1	0	0	1	PT057
	c.1223G>A	VUS	1	1	0	0	PT009
NOTCH3 (NM_000435.3)	c.2581G>C	VUS	1	1	0	0	PT129
	c.3691C>T	LB	1	0	0	1	PT077
NPC1 (NM_000271.5)	c.3206T>A (HET)	VUS	1	0	0	1	PT133
NPC2 (NM_006432)	c.441+1G>A (HOM)	LB	1	0	0	1	PT126

NR4A2 (NM_006186.4)	c.1412A>C	VUS	1	1	0	0	PT148
OPTN (NM_001008211.1)	c.476G>T	VUS	1	0	0	1	PT003
PSEN1 (NM_000021.4)	c.323G>A	VUS	2	0	0	2	PT003, PT069
	c.1198G>A	VUS	1	1	0	0	PT019
PDGFRB (NM_002609.4)	c.3296C>T	VUS	1	0	0	1	PT001
RNF216 (NM_207111.4)	c.1224+2C>T (HET)	VP	1	1	0	0	PT093
SETX (NM_001351528.2)	C.967A>G (HOM)	VUS	1	0	0	1	PT077
	C.82G>A (HET)	VUS	1	1	0	0	PT048
	C.2479A>G (HET)	LB	1	0	0	1	PT013
	c.3229G>A (HET)	LB	1	0	1	0	PT130
	C.7369C>T (HET)	B	1	0	0	1	PT012
SETX (NM_015046)	c.6842+15T>C	LB	1	0	1	0	PT006
SORL1 (NM_003105.5)	c.3050-13G>A	VUS	1	1	0	0	PT088
	c.5110G>A	LB					
TBK1 (NM_013254.4)	c.1277G>A	VUS	1	0	0	1	PT094
THAP1 (NM_018105.2)	c.523C>T	VUS	1	1	0	0	PT032
TREM2 (NM_001271821.2)	c.606G>T	LB	1	1	0	0	PT067
VPS13C (NM_020821.3)	c.5726A>T (HET)	VUS	1	0	0	1	PT102
	c.7528C>T (HET)	VUS	1	0	0	1	PT153
	c.1126T>C (HET)	VUS	2	0	0	2	PT086, PT016
	**VUS = variant of unknown significance, LB = likely benign variant, B = benign variant, HET = Heterozygous variant, HOM = Homozygous variant**						
